# Development and Validation of a Nomogram for Predicting Postoperative Delirium in Patients With Elderly Hip Fracture Based on Data Collected on Admission

**DOI:** 10.3389/fnagi.2022.914002

**Published:** 2022-06-16

**Authors:** Yin Yang, Tianpei Wang, Hua Guo, Ye Sun, Junjun Cao, Peng Xu, Yongsong Cai

**Affiliations:** ^1^Department of Orthopaedics, Xi’an Central Hospital, Xi’an Jiaotong University Health Science Center, Xi’an, China; ^2^Graduate School, Shaanxi University of Traditional Chinese Medicine, Xianyang, China; ^3^Department of Joint Surgery, Xi’an Honghui Hospital, Xi’an Jiaotong University Health Science Center, Xi’an, China

**Keywords:** postoperative delirium, elderly hip fracture, nomogram, prediction model, dementia, chronic obstructive pulmonary disease, albumin

## Abstract

Delirium is a common postoperative complication in elderly hip fracture patients that seriously affects patients’ lives and health, and early delirium risk prediction, and targeted measures can significantly reduce the incidence of delirium. The purpose of this study was to develop and validate a nomogram for the prediction of postoperative delirium (POD) in elderly hip fracture patients. A total of 328 elderly patients with hip fractures enrolled retrospectively in department 1 of our hospital were randomly divided into the training set (*n* = 230) and the internal validation set (*n* = 98). The least absolute shrinkage and selection operator (LASSO) regression analysis was used for feature variable selection, and multivariate logistic regression with a backward stepwise method was used to construct a nomogram in the training set. The discrimination efficacy and calibration efficacy of the nomogram were evaluated through the receiver operating characteristic (ROC) curve and calibration curve, respectively. The clinical usefulness was estimated through decision curve analysis (DCA) and clinical impact curve (CIC) analysis. Another validation set from department 2 of our hospital, containing 76 elderly patients with hip fractures, was used for external validation of the nomogram. A total of 43 (13.1%) and 12 (15.8%) patients had POD in department 1 and department 2, respectively. The nomogram was constructed by three predictors, including dementia, chronic obstructive pulmonary disease (COPD), and albumin level. The nomogram showed good discrimination efficacy and calibration efficacy, with the AUC of 0.791 (95% CI, 0.708–0.873), 0.820 (95% CI, 0.676–0.964), and 0.841 (95% CI, 0.717–0.966) in the training set, the internal validation set, and the external validation set, respectively. Both DCA and CIC demonstrated that this nomogram has good clinical usefulness. The nomogram constructed by dementia, COPD, and albumin level can be conveniently used to predict POD in patients with elderly hip fractures.

## Introduction

Delirium is a common post operative neuropsychiatric complication in elderly hip fracture patients, which usually occurs from a few hours to a few days after surgery and is mainly manifested as attention disorders, a decline in consciousness, and thinking disorders (Hu et al., 2022). In elderly patients with hip fractures, the incidence of post operative delirium (POD) ranges from 13% to 70%, and POD has higher mortality within 6 months (Bruce et al., 2007; Mosk et al., 2017). Additionally, POD is associated with alonger recovery time of the patients’ somatic functions, higher morbidity of pulmonary and cardiac complications, higher incidence of adverse events such as falling out of bed and unplanned extubation, a longer length of hospital stay, ICU admission, and higher economic burden on patients and their families (Bellelli et al., 2014; Krogseth et al., 2014; Gleason et al., 2015; Gottschalk et al., 2015; Zywiel et al., 2015; Mosk et al., 2017). Hence, elderly patients with hip fractures at high risk of delirium require a higher level of medical care and more careful nursing than those at low risk.

Studies have demonstrated that 30%–40% of delirium cases can be prevented, and applying targeted measures have a positive effect on reducing the incidence of delirium and the harm caused by delirium (Olofsson et al., 2018; Wang G. et al., 2021; Wang Y. et al., 2021). At present, the diagnosis of POD is mainly based on clinical observation (Inouye et al., 2014). Studies found that not all POD can be noticed in time. Hypoactive POD, which accounts for up to 71% of POD, is not easy to notice because of the non-typical symptoms (Hayhurst et al., 2020; Lee-Archer et al., 2021; Hu et al., 2022). Therefore, it is crucial to find an effective POD prediction method and early identification of elderly patients with hip fractures at high risk of POD.

In recent years, many risk factors related to POD in elderly patients with hip fractures have been found in some clinical studies, such as albumin, stroke, C-reactive protein (CRP), surgery duration, and preoperative cognitive impairment, and some of them have been used to establish prediction models of POD (Guo et al., 2016; Zhang et al., 2019). However, the prediction efficiency of each prediction model varies greatly, and there is no unified model for predicting the risk of POD after hip fracture.

In the current study, we collected the clinical data from elderly patients with hip fractures, used the least absolute shrinkage and selection operator (LASSO) regression analysis to select the predictors, and established a predictive model using logistic regression and visualized it as anomogram. Finally, we validated the prediction model in an internal validation set and an external validation set.

## Methods

### Patients

All patients with hip fractures who were treated in the Department 1 of Orthopedics of Xi’an Central Hospital from February 1, 2017, to November 1, 2021, were reviewed. The patients’ information, clinical data, and laboratory examination results on admission were collected. The inclusion criteria were: (i) age ≥ 60 years; (ii) diagnosis of hip fracture combined with imaging and clinical symptoms, including subtrochanteric fracture, femoral neck fracture, and intertrochanteric fracture; and (iii) surgical treatment including replacement, “closed reduction and internal fixation” and “open reduction internal fixation”. The exclusion criteria were as follows: (i) delirium existed before the operation or admission; (ii) long-term coma or could not cooperate with the delirium assessment; and (iii) data missing.

The data for external validation were collected in another department of Orthopedics of Xi’an Central Hospital using the same inclusion and exclusion criteria from August 1, 2020, to November 1, 2021. This study was approved by the human research ethics committee of the Xi’an Central Hospital and all procedures performed in this study were in accordance with the 1964 Declaration of Helsinki.

### Data Collection

Two investigators applied the eligibility criteria independently to review all the elderly hip fracture patients. Demographic variables, clinical data, and laboratory examination results on admission were collected, and any inconsistencies were resolved by checking the data again.

(1) Demographic variables: age, sex, body mass index (BMI).
(2) Clinical data: coronary heart disease, hypertension, cerebral infarction, dementia, diabetes, pulmonary infection, chronic obstructive pulmonary disease (COPD), duration of surgery, blood loss, American Society of Anesthesiologists Physical Status Classification (ASA class).
(3) Laboratory examination results: red blood cell count, hemoglobin, platelet (PLT) count, blood creatinine (CREA), blood urea nitrogen (BUN), serum albumin (ALB), serum globulin (GLB), glutamic-pyruvic transaminase, blood glucoselevel, and blood electrolyte (including sodium, potassium ion, and calcium ion concentrations).

### Definition of POD and POD Superimposed on Dementia

POD was diagnosed by the researcher and a psychiatrist by extracting the characteristic words of delirium in the patient’s medical record according to the Confusion Assessment Method (CAM) scale (Inouye et al., 1990), combined with the actual description of the delirium or delirium symptoms in the medical record. The scale contains four criteria: (1) acute onset or fluctuating course; (2) inattention; (3) disorganized thinking; (4) altered level of consciousness. When indicators (1) and (2) are met at the same time, or (3) combined with any one of them, or if (4) alone, then the patient can be diagnosed as POD. Accordingly, patients were divided into a POD group and a non-POD group. Elderly patients with dementia are more likely to develop delirium when hospitalized, and their prior cognitive impairment makes it more difficult to establish delirium (Lee et al., 2017). Dementia patients were defined as those with dementia before admission or operation, and those who were both dementia and POD established by the CAM scale were POD superimposed on dementia.

### Construction and Validation of the Prediction Model

In department 1, 70% of the included patients were randomly selected as the training set, and the other 30% were selected as the test set (internal validation set). The included patients in another department of Orthopedics (department 2) were selected as the external validation set. The training set was used to establish the prediction model of POD in elderly hip fracture patients, and the test set and the external validation set were used to validate the model. To avoid multicollinearity between various variables, the least absolute shrinkage and selection operator (LASSO) regression analysis with 10-fold cross-validation was conducted to screen the most useful predictive variables using the “glmnet” R package. The logistic regression model was constructed using the screened out variables, with POD as the prediction. The step wise backward method based on the likelihood-ratio test with Akaike information criterion (AIC) was applied to select the optimal model. Then, the “rms” R package was used to visualize the model and draw the nomogram. Finally, the ROC curves, calibration curves, decision curve (DCA), and clinical impact curve (CIC) were applied to assess the nomogram’s prediction accuracy.

### Statistical Analysis

Continuous variables were analyzed by independent Student’s t-test or Mann-Whitney U and presented as the mean (standard deviation) or median (quartile interval). Categoricalvariables were analyzed according to the distribution using the chi-square test, Wilcoxon rank-sum test, Fisher’s test, and continuity correction and presented as case number and composition ratio. Lasso regression analyses and multivariate logistic regression analyses were used. The statistical analyses were performed by SPSS 26.0, and a *p-*value less than 0.05 was considered statistically significant.

## Results

### Patient Demographic and Clinical Information

A total of 619 patients who under went surgical treatment for hip fracture were screened, and 404 of them met the inclusion criteria. Of the included 404 patients, 328 patients were from department 1 of orthopedics of Xi’an Central Hospital and 76 patients from another department (department 2). Patient characteristics in the two departments are listed in Table 1. No significant difference (*p* = 0.669) in the proportion of POD was found between the two departments, and the proportions were 13.1% and 15.8%, respectively. Some distribution of patient characteristics in these two departments was different. Compared with department 1, the proportions of dementia, pulmonary infection, and COPD was higher, the level of albumin was lower, and the operative duration was longer in the patients in department 2. In department 1, 70% of the included patients (*n* = 230) were divided into the training set, and the other 30% (*n* = 98) were selected as the internal validation set. The patient characteristics in the training set are shown in Table 2. We also summarized the characteristics of patients in internal and external validation sets (Supplementary Table 1 and Supplementary Table 2).

**Table 1:** Patient characteristics of the department 1 and department 2.

		Department 1 (*n* = 328)	Department 2 (*n* = 76)	*p*
Delirium (%)	no	285 (86.9)	64 (84.2)	0.669
	yes	43 (13.1)	12 (15.8)
Age [mean (SD)]		81.85 (7.73)	80.61 (8.70)	0.218
BMI [median (IQR)]		22.00 [19.00, 24.00]	22.00 [19.00, 24.25]	0.548
Sex (%)	male	95 (29.0)	23 (30.3)	0.933
	female	233 (71.0)	53 (69.7)
Hypertension (%)	no	147 (44.8)	35 (46.1)	0.946
	yes	181 (55.2)	41 (53.9)
CHD (%)	no	226 (68.9)	44 (57.9)	0.089
	yes	102 (31.1)	32 (42.1)
Diabetes (%)	no	251 (76.5)	55 (72.4)	0.54
	yes	77 (23.5)	21 (27.6)
Cerebral infarction (%)	no	244 (74.4)	54 (71.1)	0.652
	yes	84 (25.6)	22 (28.9)
Dementia (%)	no	305 (93.0)	63 (82.9)	0.01
	yes	23 (7.0)	13 (17.1)
Pulmonary infection (%)	no	272 (82.9)	53 (69.7)	0.014
	yes	56 (17.1)	23 (30.3)
COPD (%)	no	322 (98.2)	64 (84.2)	<0.001
	yes	6 (1.8)	12 (15.8)
ASA (%)	0	1 (0.3)	0 (0.0)	1
	≥1	327 (99.7)	76 (100.0)
Na^+^ concentration [median (IQR)]		139.00 [137.00, 142.00]	139.00 [137.00, 141.00]	0.665
K^+^ concentration [mean (SD)]		3.93 (0.48)	3.97 (0.45)	0.5
Ca2^+^ concentration [mean (SD)]		2.23 (0.19)	2.23 (0.15)	0.976
ALB [mean (SD)]		37.80 (5.03)	36.46 (3.90)	0.03
Globulin [median (IQR)]		25.80 [22.37, 29.50]	26.60 [24.15, 30.60]	0.051
ALT [mean (SD)]		17.95 (15.90)	18.57 (21.17)	0.778
BUN [median (IQR)]		7.00 [5.00, 9.00]	6.00 [5.00, 8.00]	0.18
CREA [median (IQR)]		67.00 [54.00, 84.25]	65.00 [53.75, 80.25]	0.253
Blood glucose [median (IQR)]		6.59 [5.68, 8.10]	6.75 [5.86, 8.43]	0.47
Erythrocyte count [mean (SD)]		3.62 (0.66)	3.52 (0.63)	0.239
Hemoglobin [mean (SD)]		111.13 (19.99)	109.93 (19.70)	0.638
PLT [median (IQR)]		175.50 [135.75, 224.25]	188.50 [149.75, 242.00]	0.155
Operative duration [mean (SD)]		104.94 (47.08)	193.17 (80.02)	<0.001
Intraoperative blood loss [median (IQR)]		100.00 [50.00, 200.00]	100.00 [50.00, 200.00]	0.5

*CHD, coronary heart disease; BMI, body mass index; COPD, chronic obstructive pulmonary disease; ALB, albumin; ALT, alanine transaminase; BUN, blood urea nitrogen; CREA, creatinine; PLT, platelet; ASA, American Society of Anesthesiologists Physical Status Classification*.

**Table 2:** Patient characteristics of the training set.

		Total (*n* = 230)	Non-POD (*n* = 200)	POD (*n* = 30)	*p*
Age [mean (SD)]		81.64 (7.65)	81.42 (7.63)	83.10 (7.77)	0.265
Sex (%)	male	68 (29.6)	5 (28.5)	11 (36.7)	0.484
	female	162 (70.4)	143 (71.5)	19 (63.3)
BMI [median (IQR)]		21.00 [19.00, 24.00]	21.50 [19.00, 24.00]	21.00 [20.00, 22.75]	0.643
Hypertension (%)	no	98 (42.6)	88 (44.0)	10 (33.3)	0.366
	yes	132 (57.4)	112 (56.0)	20 (66.7)
CHD (%)	no	157 (68.3)	137 (68.5)	20 (66.7)	1
	yes	73 (31.7)	63 (31.5)	10 (33.3)
Cerebral infarction (%)	no	167 (72.6)	145 (72.5)	22 (73.3)	1
	yes	63 (27.4)	55 (27.5)	8 (26.7)
Dementia (%)	no	214 (93.0)	190 (95.0)	24 (80.0)	0.009
	yes	16 (7.0)	10 (5.0)	6 (20.0)
Pulmonary infection (%)	no	189 (82.2)	165 (82.5)	24 (80.0)	0.798
	yes	41 (17.8)	35 (17.5)	6 (20.0)
COPD (%)	no	226 (98.3)	198 (99.0)	28 (93.3)	0.084
	yes	4 (1.7)	2 (1.0)	2 (6.7)
ASA (%)	0	1 (0.4)	1 (0.5)	0 (0.0)	1
	≥1	229 (99.6)	199 (99.5)	30 (100.0)
Diabetes (%)	no	178 (77.4)	155 (77.5)	23 (76.7)	1
	yes	52 (22.6)	45 (22.5)	7 (23.3)
Na^+^ concentration [median (IQR)]		139.00 [137.00, 141.00]	139.00 [137.00, 141.00]	140.50 [137.50, 141.75]	0.546
K^+^ concentration [mean (SD)]		3.94 (0.49)	3.94 (0.48)	3.89 (0.53)	0.609
Ca2^+^ concentration [mean (SD)]		2.24 (0.19)	2.23 (0.17)	2.26 (0.31)	0.51
ALB [mean (SD)]		37.56 (4.69)	38.13 (4.51)	33.73 (4.02)	<0.001
Globulin [median (IQR)]		25.70 [22.33, 28.60]	25.80 [22.48, 28.60]	24.80 [21.65, 29.20]	0.451
ALT [mean (SD)]		17.83 (16.63)	18.27 (17.65)	14.90 (6.09)	0.302
BUN [median (IQR)]		7.00 [5.00, 9.00]	7.00 [5.00, 9.00]	7.00 [6.00, 9.75]	0.634
CREA [median (IQR)]		69.00 [57.00, 85.00]	69.00 [57.00, 85.25]	72.50 [54.00, 82.00]	0.771
Blood glucose [median (IQR)]		6.56 [5.59, 8.21]	6.51 [5.58, 8.13]	6.98 [5.72, 8.60]	0.663
Erythrocyte count [mean (SD)]		3.58 (0.61)	3.57 (0.62)	3.67 (0.49)	0.375
Hemoglobin [mean (SD)]		111.00 (19.37)	110.58 (19.98)	113.73 (14.61)	0.408
PLT [median (IQR)]		172.50 [136.25, 219.50]	175.00 [136.50, 220.25]	166.50 [136.75, 214.00]	0.877
Operative duration [mean (SD)]		102.36 (40.49)	103.38 (41.91)	95.60 (28.88)	0.328
Intraoperative blood loss [median (IQR)]		100.00 [50.00, 200.00]	100.00 [50.00, 200.00]	100.00 [50.00, 137.50]	0.412

*CHD, coronary heart disease; BMI, body mass index; COPD, chronic obstructive pulmonary disease; ALB, albumin; ALT, alanine transaminase; BUN, blood urea nitrogen; CREA, creatinine; PLT, platelet; ASA, American Society of Anesthesiologists Physical Status Classification*.

### Feature Selection and Prediction Model Construction

**Figure 1:**
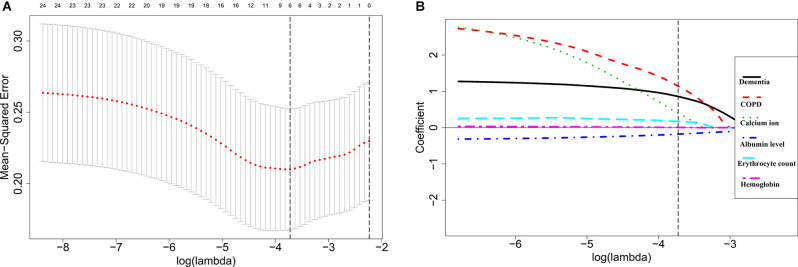
Selection of feature variables using the least absolute shrinkage and selection operator (LASSO) regression analysis. **(A)** Cross-validation for the feature variable selection of optimal lambda value in the LASSO model. The left and right dotted vertical lines represent the values of log (lambda.min) and log (lambda.1se). **(B)** LASSO coefficient profiles of the six feature variables. The dotted vertical line represents the value of log (lambda.min).

**Figure 2:**
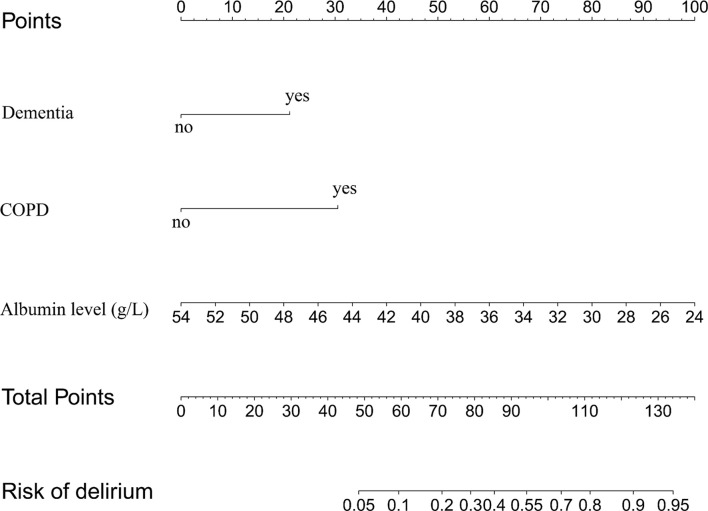
The nomogram predicts the risk of POD in elderly patients with hip fractures, based on dementia, COPD, and ALB levels. COPD, chronic obstructive pulmonary disease; ALB, albumin.

A total of 26 variables were extracted from each patient. To identify the key variables associated with POD in elderly hip fracture patients, 26 variables were used to construct a LASSO model in the training set. The results showed that when the lambda value was selected as lambda.min (0.02409), a total of six variables with nonzero coefficients were screened out (Figures 1A,B). The six variables were further identified by stepwise backward logistic regression analysis, and three of them (dementia, COPD, and serum albumin level) were screened for optimal prediction model establishment. The model was established and visualized as a nomogram (Figure 2 and Table 3).

**Table 3:** Results of the multivariable logistic analysis.

Predictor	Coefficient	SE	OR (95% CI)	*p*
Dementia	1.453	0.632	4.275 (1.240–14.742)	0.021
COPD	2.097	1.054	8.14 (1.031–64.241)	0.047
ALB	−0.229	0.054	0.795 (0.716–0.884)	0.00002

*COPD, chronic obstructive pulmonary disease; ALB, albumin*.

### POD Nomogram Evaluation

**Figure 3:**
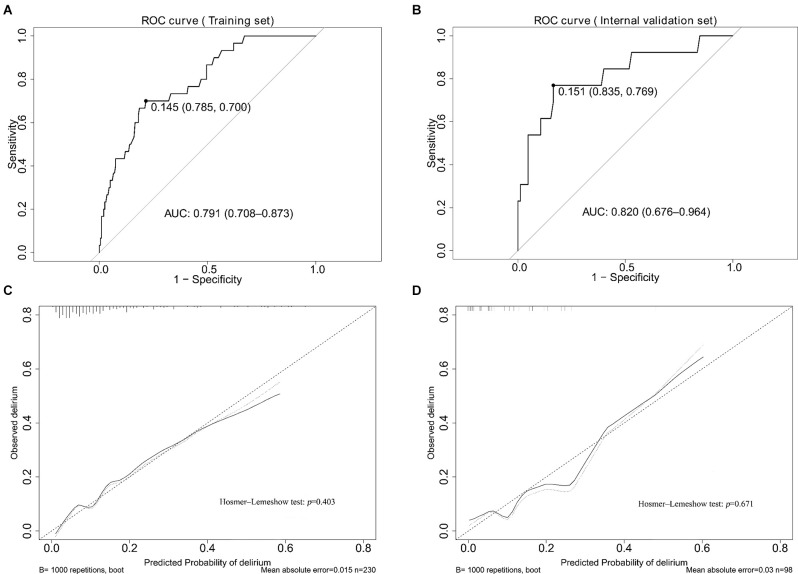
The results of the ROC curve and calibration analysis of the nomogram in the training set and the internal validation set. **(A,B)** The AUC and the calibration curve of the nomogram for predicting POD in the training set. **(C,D)** The AUC and the calibration curve of the nomogram for predicting POD in the internal validation set.

**Figure 4:**
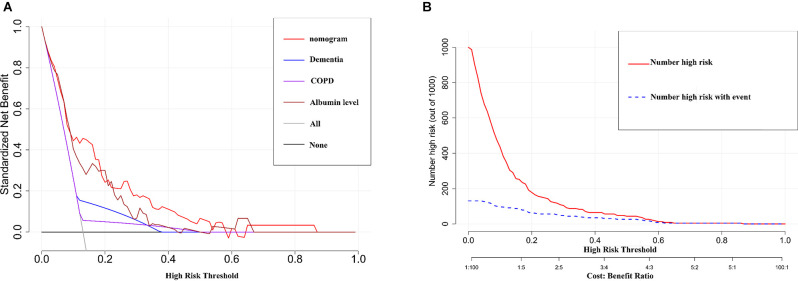
The results of the DCA and the CIC analysis of the nomogram in the training set. **(A)** The DCA curve of the nomogram for predicting POD. It revealed that the nomogramcould obtain a greater net benefit than either the “treat all” or the “treat none” strategy and also get a higher net benefit than each predictor alone. **(B)** The CIC curve of the nomogram for predicting POD. The solid red line (Number high risk) represents the number of POD patients predicted using the nomogramat each threshold probability; the dotted blue line (number high risk with event) represents the number of true-positive POD patients at each threshold probability.

**Figure 5:**
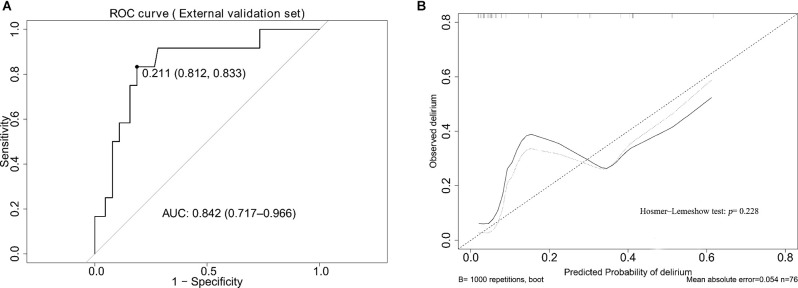
The results of the ROC curve and calibration analysis of the nomogram in the external validation set. **(A)** The AUC of the nomogram for predicting POD in the external validation set. **(B)** The calibration curve of the nomogram in the external validation set.

The AUCs for the nomogram were 0.791 (95% CI, 0.708–0.873) and 0.820 (95% CI, 0.676–0.964) in the training set and the internal validation set, respectively (Figures 3A,B, Supplementary Table 3). The calibration curves of the present predictive model showed good agreement between prediction and observation in the training set and the internal validation set, and the Hosmer–Lemeshow test showed a non-significant p-value of 0.403 and 0.671, respectively (Figures 3C,D). DCA curve and CIC curve were used to evaluate the clinical usefulness of the present nomogram. The DCA curve showed that if the threshold probability was with in the range from 0.05 to 0.6, this prediction model could obtain a greater net benefit than either the “treat all” or the “treat none” strategy and also get a higher net benefit than each predictor alone (Figure 4A). The CIC result showed that the number of patients who were at high risk (the number of POD predicted using the nomogram) was highly matched with the number of patients who were at high risk with the event (the number of true-positive POD) when the threshold probability was above 0.6 (Figure 4B). In the external validation set, the AUC for the nomogram was 0.841 (95% CI, 0.717–0.966; Figure 5A). In addition, the calibration curve of the nomogram also showed a good agreement between prediction and observation in the external validation set, and the *p*-value of the Hosmer–Lemeshow test was 0.228 (Figure 5B). The results showed that the nomogram had a good predictive value for POD in elderly patients with hip fractures.

## Discussion

In this study, we developed and validated a novel nomogram model for POD in elderly hip fracture patients. The nomogram was constructed by three variables, including dementia, COPD, and serum albumin level. The results indicated that the nomogram had good discrimination efficacy and calibration efficacy, and showed good clinical usefulness.

The clinical data of 404 patients were included in the present study. The incidence of POD in elderly hip fracture patients was 13.6% (55/404), which was consistent with Zhang’s study (14.3%; Zhang et al., 2019) and Zhao’s study (12.2%; Zhao et al., 2021), but lower than Wang’s study (19.12%; Wang Y. et al., 2021) and Guo’s study (21%; Guo et al., 2016). This may be related to delirium diagnostic strategies. All of the above studies, including the present one, were based on retrospective searches in clinical records for keywords associated with the delirium diagnostic method. It is known that the retrospective search for elements of delirium in the medical record is associated with significant errors in the estimation of the delirium occurrence since it usually underestimates patients with hypoactive delirium. Medical staff, especially non-neuropsychiatrists or non-geriatricians, are always attracted by the hyperactive delirium and mixed delirium, and ignored the hypoactive delirium. Our previous study found that dementia history, low albumin levels, and intraoperative hypotension were the independent risk factors related to POD in elderly patients with hip fractures [Wang et al., 2022 (Chinese)]. However, the assessment criteria of intraoperative hypotension were subjective and different doctors may obtain different results, so the nomogram model contained the factor of intraoperative hypotension did not have good stability. In this study, all the three predictors, including dementia, COPD, and albumin, were easy to obtain objectively and reliably. Many studies have explored the risk factors of POD in elderly hip fractures and established prediction models (Guo et al., 2016; Zhang et al., 2019). Zhang et al. (2019) found that preoperative cognitive impairment, ASA, multivariate medical comorbidities, intensive care, and transfusion exceeding 2 units of red blood cell were the independent predictors of POD, and the C-index of the prediction model constructed by the five predictors was 0.67 (0.62–0.72), which did not have a good discrimination efficacy for POD. In our study, the AUC of the nomogram for predicting POD in elderly hip fracture was 0.791, 0.820, and 0.841 in the training set, internal validation set, and external validation set, respectively. Six of the 30 patients with POD (20%) had a history of dementia in the training set. Multivariate logistic regression showed thata history of dementia was a risk factor for POD in elderly hip fracture patients (OR = 4.27, 95% CI: 1.24–14.74), suggesting that patients with dementia are more likely to develop delirium, which was consistent with previous studies. Mosk et al. (2017) found that elderly hip fracture patients were vulnerable to delirium, especially in those with hip fracture and dementia, with the incidence of delirium as high as 57.7%. Another study found that the risk of delirium in patients with cognitive decline/dementia was 3.70 times higher than that of patients without cognitive decline/dementia (Ding et al., 2021). This may be related to the fact that patients with dementia often have cognitive impairment and degeneration of the nervous system, which makes them more likely to develop delirium. Focusing on hip fracture patients with dementia and seeking psychiatric assistance may help reduce the incidence of delirium (Davis et al., 2019).

Hypoalbuminemia was another risk factor for POD in patients with hip fracture in our study, which was consistent with Chu’s study (Chu et al., 2021) and Kong’s study (Kong et al., 2022). Chu et al. found that albumin ≤32.26 g/L was an independent risk factor in elderly patients with hip fracture, which was slightly different from Kong’s study (albumin ≤32.26 g/L vs. albumin ≤40 g/L). In elderly patients with hip fractures, hypoalbuminemia may result from blood loss from trauma, insufficient food intake, stress reaction, and other reasons. The mechanism of hypoalbuminemia leads to delirium is unclear, it may be the albumin can not only transport a variety of trace elements and drugs but also has the functions of antioxidation, scavenging free radicals, and protecting the microcirculation, playing a vital role in the body’s metabolism (Quinlan et al., 2005). Clinical prevention targeted low albumin levels in elderly patients with hip fractures may play a role in reducing the risk of POD (Trzepacz and Francis, 1990).

COPD is the third leading cause of death in the world, especially in elderly patients (Vestbo et al., 2013). In our study, COPD was one of the three predictors of the nomogram (OR = 8.14, 95% CI: 1.03–64.24, *p* = 0.046). Cui et al. (2019) conducted a retrospective study on risk factors related to POD in elderly patients with spinal operation and found that COPD was an independent risk factor of POD. Similar results had been found in other studies. Brunna et al. found that COPD increased the risk of POD during hospitalization of elderly fracture patients (Lima et al., 2021). COPD had also been identified as a risk factor for POD in other diseases, such as coronary artery bypass grafting (CABG; Szylińska et al., 2020).

Some potential limitations should be taken into account when interpreting these results. First, this research was a retrospective study, and POD was diagnosed by extracting the keywords associated with the CAM scale from the patient’s medical record, which usually underestimated patients with hypoactive delirium; Second, the sample size included in the study was insufficient, especially in the internal validation set and the external validation set, and the evaluation of the results may be biased; Third, although COPD and dementia were identified as POD risk factors and used to construct the nomogram, there were only 16 patients with dementia and four with COPD in the training set, which may limit the clinical applicability; Lastly, although we used an external validation set to verify the results in our study, this external validation set came from another department of the same hospital, so it did not have good external applicability.

## Conclusion

Dementia, COPD, and serum albumin levels were the key predictors for POD in elderly patients with hip fractures. The nomogram model constructed based on these predictors had great clinical application and provided a visual tool for the diagnosis of POD in elderly patients with hip fractures. Further prospective studies should be conducted to confirm our results.

## Data Availability Statement

The original contributions presented in the study are included in the article/Supplementary Material, further inquiries can be directed to the corresponding author/s.

## Ethics Statement

The studies involving human participants were reviewed and approved by the human research ethics committee of the Xi’an Central Hospital. Written informed consent was not provided because this research was a retrospective study.

## Author Contributions

PX and YC designed the study. YY, TW, YS, and JC collected the data and carried out the data analysis. HG and YS drew the figures. TW, YC, and YY drafted the manuscript. PX, HG, and JC participated in modifying the manuscript. All authors contributed to the article and approved the submitted version.

## Conflict of Interest

The authors declare that the research was conducted in the absence of any commercial or financial relationships that could be construed as a potential conflict of interest.

## Publisher’s Note

All claims expressed in this article are solely those of the authors and do not necessarily represent those of their affiliated organizations, or those of the publisher, the editors and the reviewers. Any product that may be evaluated in this article, or claim that may be made by its manufacturer, is not guaranteed or endorsed by the publisher.
