# Supplementary material for: Development and Validation of a Nomogram for Predicting Postoperative Delirium in Patients With Elderly Hip Fracture Based on Data Collected on Admission
Source: Front Aging Neurosci. 2022 Jun 16;14:914002. doi: 10.3389/fnagi.2022.914002 (PMC9243358; doi:10.3389/fnagi.2022.914002)
Supplement: Supplementary file 1 [file Table_1.DOCX]

**Table 1 Patient characteristics of the internal validation set**

|  |  | Total (n=98) | Non-POD (n=85) | POD (n=13) | *p* |
| --- | --- | --- | --- | --- | --- |
| Age (mean (SD)) | | 82.34 (7.94) | 82.47 (7.69) | 81.46 (9.71) | 0.672 |
| Sex (%) | male | 27 ( 27.6) | 23 ( 27.1) | 4 ( 30.8) | 0.749 |
|  | female | 71 ( 72.4) | 62 ( 72.9) | 9 ( 69.2) | |
| BMI (median [IQR]) | | 22.00 [18.25, 24.00] | 22.00 [19.00, 24.00] | 19.00 [17.00, 24.00] | 0.122 |
| Hypertension (%) | no | 49 ( 50.0) | 43 ( 50.6) | 6 ( 46.2) | 1 |
|  | yes | 49 ( 50.0) | 42 ( 49.4) | 7 ( 53.8) | |
| CHD (%) | no | 69 ( 70.4) | 61 ( 71.8) | 8 ( 61.5) | 0.518 |
|  | yes | 29 ( 29.6) | 24 ( 28.2) | 5 ( 38.5) | |
| Cerebral infarction (%) | no | 77 ( 78.6) | 69 ( 81.2) | 8 ( 61.5) | 0.144 |
|  | yes | 21 ( 21.4) | 16 ( 18.8) | 5 ( 38.5) | |
| Dementia (%) | no | 91 ( 92.9) | 81 ( 95.3) | 10 ( 76.9) | 0.047 |
|  | yes | 7 ( 7.1) | 4 ( 4.7) | 3 ( 23.1) | |
| Pulmonary infection (%) | no | 83 ( 84.7) | 72 ( 84.7) | 11 ( 84.6) | 1 |
|  | yes | 15 ( 15.3) | 13 ( 15.3) | 2 ( 15.4) | |
| COPD (%) | no | 96 ( 98.0) | 84 ( 98.8) | 12 ( 92.3) | 0.249 |
|  | yes | 2 ( 2.0) | 1 ( 1.2) | 1 ( 7.7) | |
| Diabetes (%) | no | 73 ( 74.5) | 62 ( 72.9) | 11 ( 84.6) | 0.506 |
|  | yes | 25 ( 25.5) | 23 ( 27.1) | 2 ( 15.4) | |
| ASA (%) | 0 | 0 | 0 | 0 | - |
|  | ≥1 | 98 (100.0) | 85 (100.0) | 13 (100.0) | |
| Na^+^ concentration (median [IQR]) | | 139.50 [137.00, 142.00] | 139.00 [137.00, 142.00] | 140.00 [136.00, 140.00] | 0.484 |
| K^+^ concentration (mean (SD)) | | 3.91 (0.47) | 3.90 (0.49) | 3.99 (0.34) | 0.529 |
| Ca2^+^ concentration (mean (SD)) | | 2.23 (0.19) | 2.22 (0.15) | 2.29 (0.35) | 0.198 |
| ALB (mean (SD)) | | 38.39 (5.74) | 39.29 (5.34) | 32.45 (4.81) | <0.001 |
| Globulin (median [IQR]) | | 26.05 [22.65, 30.28] | 26.30 [22.30, 30.00] | 25.40 [23.40, 31.60] | 0.565 |
| ALT (mean (SD)) | | 18.25 (14.10) | 18.10 (14.23) | 19.23 (13.71) | 0.789 |
| BUN (median [IQR]) | | 6.00 [5.00, 8.00] | 6.00 [5.00, 8.00] | 6.00 [5.00, 9.00] | 0.811 |
| CREA (median [IQR]) | | 62.00 [50.25, 82.75] | 61.00 [50.00, 84.00] | 67.00 [54.00, 81.00] | 0.933 |
| Blood glucose (median [IQR]) | | 6.63 [5.95, 7.81] | 6.64 [5.95, 7.96] | 6.53 [5.93, 6.99] | 0.568 |
| Erythrocyte count (mean (SD)) | | 3.72 (0.78) | 3.74 (0.68) | 3.57 (1.26) | 0.455 |
| Hemoglobin (mean (SD)) | | 111.44 (21.47) | 113.40 (21.01) | 98.62 (20.73) | 0.02 |
| PLT (median [IQR]) | | 179.00 [132.75, 241.50] | 179.00 [131.00, 238.00] | 181.00 [150.00, 254.00] | 0.806 |
| Operative duration (mean (SD)) | | 111.00 (59.57) | 106.38 (54.07) | 141.23 (84.16) | 0.049 |
| Intraoperative blood loss (median [IQR]) | | 100.00 [50.00, 200.00] | 100.00 [50.00, 200.00] | 100.00 [50.00, 200.00] | 0.489 |

CHD, coronary heart disease; BMI, body mass index; COPD, chronic obstructive pulmonary disease; ALB, albumin; ALT, alanine transaminase; BUN, blood urea nitrogen; CREA, creatinine; PLT, platelet; ASA, American Society of Anesthesiologists Physical Status Classification.
