# Supplementary material for: Development and Validation of a Nomogram for Predicting Postoperative Delirium in Patients With Elderly Hip Fracture Based on Data Collected on Admission
Source: Front Aging Neurosci. 2022 Jun 16;14:914002. doi: 10.3389/fnagi.2022.914002 (PMC9243358; doi:10.3389/fnagi.2022.914002)
Supplement: Supplementary file 2 [file Table_2.DOCX]

**Table 2 Patient characteristics of the external validation set**

|  |  | Total (n=76) | Non-POD (n=64) | POD (n=12) | *p* |
| --- | --- | --- | --- | --- | --- |
| Age (mean (SD)) | | 80.61 (8.70) | 80.05 (8.53) | 83.58 (9.36) | 0.198 |
| Sex (%) | male | 23 ( 30.3) | 19 ( 29.7) | 4 ( 33.3) | 1 |
|  | female | 53 ( 69.7) | 45 ( 70.3) | 8 ( 66.7) | |
| BMI (median [IQR]) | | 22.00 [19.00, 24.25] | 22.00 [20.00, 25.00] | 19.00 [18.00, 21.25] | 0.023 |
| Hypertension (%) | no | 35 ( 46.1) | 28 ( 43.8) | 7 ( 58.3) | 0.529 |
|  | yes | 41 ( 53.9) | 36 ( 56.2) | 5 ( 41.7) | |
| CHD (%) | no | 44 ( 57.9) | 39 ( 60.9) | 5 ( 41.7) | 0.34 |
|  | yes | 32 ( 42.1) | 25 ( 39.1) | 7 ( 58.3) | |
| Cerebral infarction (%) | no | 54 ( 71.1) | 43 ( 67.2) | 11 ( 91.7) | 0.162 |
|  | yes | 22 ( 28.9) | 21 ( 32.8) | 1 ( 8.3) | |
| Dementia (%) | no | 63 ( 82.9) | 58 ( 90.6) | 5 ( 41.7) | <0.001 |
|  | yes | 13 ( 17.1) | 6 ( 9.4) | 7 ( 58.3) | |
| Pulmonary infection (%) | no | 53 ( 69.7) | 45 ( 70.3) | 8 ( 66.7) | 1 |
|  | yes | 23 ( 30.3) | 19 ( 29.7) | 4 ( 33.3) | |
| COPD (%) | no | 64 ( 84.2) | 57 ( 89.1) | 7 ( 58.3) | 0.018 |
|  | yes | 12 ( 15.8) | 7 ( 10.9) | 5 ( 41.7) | |
| ASA (%) | 0 | 0 | 0 | 0 | - |
|  | ≥1 | 76 (100.0) | 64 (100.0) | 12 (100.0) | |
| Diabetes (%) | no | 55 ( 72.4) | 45 ( 70.3) | 10 ( 83.3) | 0.492 |
|  | yes | 21 ( 27.6) | 19 ( 29.7) | 2 ( 16.7) | |
| Na^+^ concentration (median [IQR]) | | 139.00 [137.00, 141.00] | 139.00 [137.75, 141.00] | 138.00 [135.75, 140.50] | 0.401 |
| K^+^ concentration (mean (SD)) | | 3.97 (0.45) | 4.01 (0.41) | 3.74 (0.55) | 0.049 |
| Ca2^+^ concentration (mean (SD)) | | 2.23 (0.15) | 2.24 (0.15) | 2.17 (0.11) | 0.14 |
| ALB (mean (SD)) | | 36.46 (3.90) | 36.91 (3.67) | 34.09 (4.38) | 0.021 |
| Globulin (median [IQR]) | | 26.60 [24.15, 30.60] | 26.60 [23.95, 29.77] | 26.40 [24.93, 31.07] | 0.765 |
| ALT (mean (SD)) | | 18.57 (21.17) | 19.17 (22.57) | 15.33 (11.13) | 0.568 |
| BUN (median [IQR]) | | 6.00 [5.00, 8.00] | 6.00 [5.00, 8.25] | 6.00 [5.00, 7.00] | 0.609 |
| CREA (median [IQR]) | | 65.00 [53.75, 80.25] | 64.00 [52.50, 81.00] | 67.50 [58.75, 75.50] | 0.554 |
| Blood glucose (median [IQR]) | | 6.75 [5.86, 8.43] | 6.75 [5.97, 8.43] | 6.38 [5.51, 8.63] | 0.608 |
| Erythrocyte count (mean (SD)) | | 3.52 (0.63) | 3.54 (0.64) | 3.43 (0.61) | 0.569 |
| Hemoglobin (mean (SD)) | | 109.93 (19.70) | 110.80 (19.83) | 105.33 (19.14) | 0.381 |
| PLT (median [IQR]) | | 188.50 [149.75, 242.00] | 188.50 [153.00, 238.75] | 200.50 [136.00, 308.25] | 0.754 |
| Operative duration (mean (SD)) | | 193.17 (80.02) | 197.39 (83.46) | 170.67 (55.77) | 0.291 |
| Intraoperative blood loss (median [IQR]) | | 100.00 [50.00, 200.00] | 100.00 [50.00, 270.00] | 100.00 [50.00, 200.00] | 0.572 |

CHD, coronary heart disease; BMI, body mass index; COPD, chronic obstructive pulmonary disease; ALB, albumin; ALT, alanine transaminase; BUN, blood urea nitrogen; CREA, creatinine; PLT, platelet; ASA, American Society of Anesthesiologists Physical Status Classification.
