# Supplementary material for: Development and Validation of a Nomogram for Predicting Postoperative Delirium in Patients With Elderly Hip Fracture Based on Data Collected on Admission
Source: Front Aging Neurosci. 2022 Jun 16;14:914002. doi: 10.3389/fnagi.2022.914002 (PMC9243358; doi:10.3389/fnagi.2022.914002)
Supplement: Supplementary file 3 [file Table_3.DOCX]

**Table 3 The discrimination efficacy of the nomogram**

|  | AUC (95% CI) | Sensitivity | Specificity | *p* |
| --- | --- | --- | --- | --- |
| Training set | 0.791 (0.708-0.873) | 0.7 | 0.785 | 1.434E-07 |
| Internal validation set | 0.820 (0.676-0.964) | 0.769 | 0.835 | 0.0001 |
| External validation set | 0.841 (0.717-0.966) | 0.833 | 0.812 | 9.479E-05 |
